# 4-Di­chloro­methyl-4-methyl-5-(nitro­meth­yl)cyclo­hex-2-enone

**DOI:** 10.1107/S1600536813027517

**Published:** 2013-10-16

**Authors:** Sladjana B. Novaković, Marko V. Rodić, Željko K. Jaćimović, Zoran Ratković, Slobodan Sukdolak

**Affiliations:** a Vinča Institute of Nuclear Sciences, Laboratory of Theoretical Physics and Condensed Matter Physics, PO Box 522, University of Belgrade, 11001 Belgrade, Serbia; b Faculty of Sciences, University of Novi Sad, Trg Dositeja Obradovića 3, 21000 Novi Sad, Serbia; c Faculty of Metallurgy and Technology, University of Montenegro, Cetinjski put bb, 81000 Podgorica, Montenegro; d Faculty of Sciences, Department of Chemistry, University of Kragujevac, R. Domanovića 12, 34000 Kragujevac, Serbia

## Abstract

In the title compound, C_9_H_11_Cl_2_NO_3_, the six-membered ring adopts a screw-chair conformation. In the crystal, two different C—H⋯O hydrogen bonds involving the same acceptor atom connect the mol­ecules into a chain extending along the *c-*axis direction.

## Related literature

For the synthetic procedure, see: Wenkert *et al.* (1969 ▶). For polyfunctionalized products obtained by similar Michael reactions with carbanions, see: Stefanović *et al.* (1983 ▶); Solujić *et al.* (1991 ▶, 1999 ▶). For a related crystal structure, see: Yang & Carter (2010 ▶).
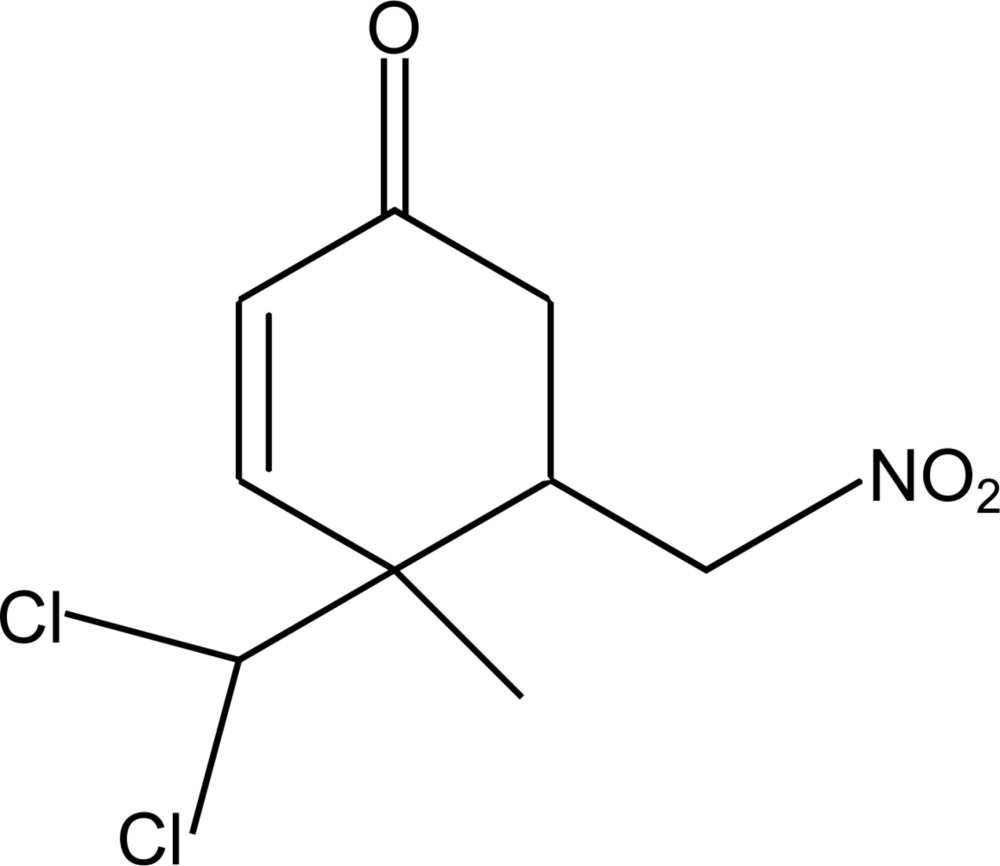


## Experimental

### 

#### Crystal data

C_9_H_11_Cl_2_NO_3_
*M* *_r_* = 252.09
Monoclinic, 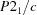

*a* = 13.8922 (7) Å
*b* = 10.4531 (9) Å
*c* = 7.8696 (5) Å
β = 101.682 (6)°
*V* = 1119.12 (13) Å^3^
*Z* = 4
Cu *K*α radiation
μ = 5.14 mm^−1^
*T* = 293 K
0.11 × 0.10 × 0.05 mm

#### Data collection

Agilent Gemini S diffractometer
Absorption correction: multi-scan (*CrysAlis PRO*; Agilent, 2013 ▶) *T*_min_ = 0.288, *T*_max_ = 1.000
4083 measured reflections
2160 independent reflections
1674 reflections with *I* > 2σ(*I*)
*R*_int_ = 0.016

#### Refinement

*R*[*F* ^2^ > 2σ(*F* ^2^)] = 0.073
*wR*(*F* ^2^) = 0.214
*S* = 1.13
2160 reflections
137 parameters
H-atom parameters constrained
Δρ_max_ = 0.44 e Å^−3^
Δρ_min_ = −0.46 e Å^−3^

### 

Data collection: *CrysAlis PRO* (Agilent, 2013 ▶); cell refinement: *CrysAlis PRO*; data reduction: *CrysAlis PRO*; program(s) used to solve structure: *SHELXS97* (Sheldrick, 2008 ▶); program(s) used to refine structure: *SHELXL97* (Sheldrick, 2008 ▶); molecular graphics: *ORTEP-3 for Windows* (Farrugia, 2012 ▶) and *Mercury* (Macrae *et al.*, 2006 ▶); software used to prepare material for publication: *WinGX* (Farrugia, 2012 ▶), *PLATON* (Spek, 2009 ▶) and *PARST* (Nardelli, 1995 ▶).

## Supplementary Material

Crystal structure: contains datablock(s) I, global. DOI: 10.1107/S1600536813027517/bt6936sup1.cif

Structure factors: contains datablock(s) I. DOI: 10.1107/S1600536813027517/bt6936Isup2.hkl

Supplementary material file. DOI: 10.1107/S1600536813027517/bt6936Isup3.cml

Additional supplementary materials:  crystallographic information; 3D view; checkCIF report

## Figures and Tables

**Table 1:** Hydrogen-bond geometry (Å, °)

*D*—H⋯*A*	*D*—H	H⋯*A*	*D*⋯*A*	*D*—H⋯*A*
C8—H8⋯O3^i^	0.98	2.24	3.189 (5)	164
C1—H1a⋯O3^i^	0.97	2.56	3.503 (6)	164

Symmetry code: (i) 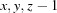.

**Fig. 1.**
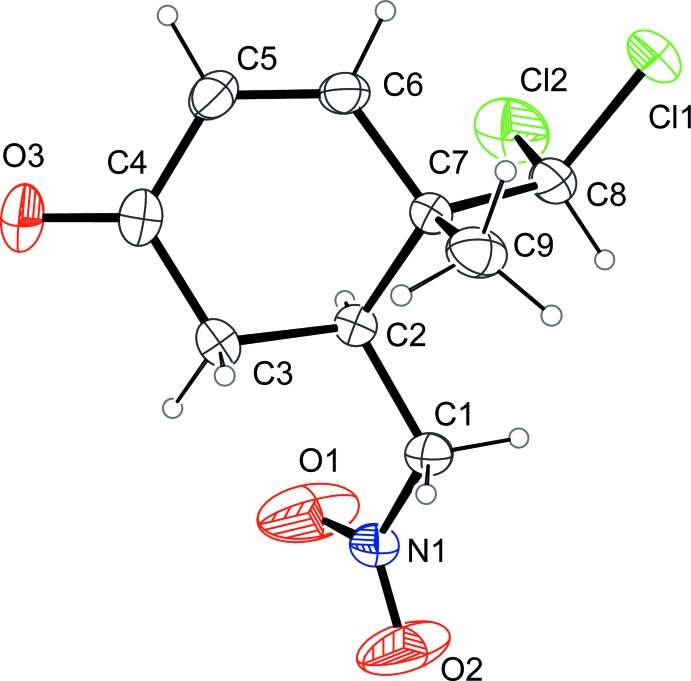
The molecular structure of the title compound, with atom labels and 30% probability displacement ellipsoids for non-H atoms.

**Fig. 2.**
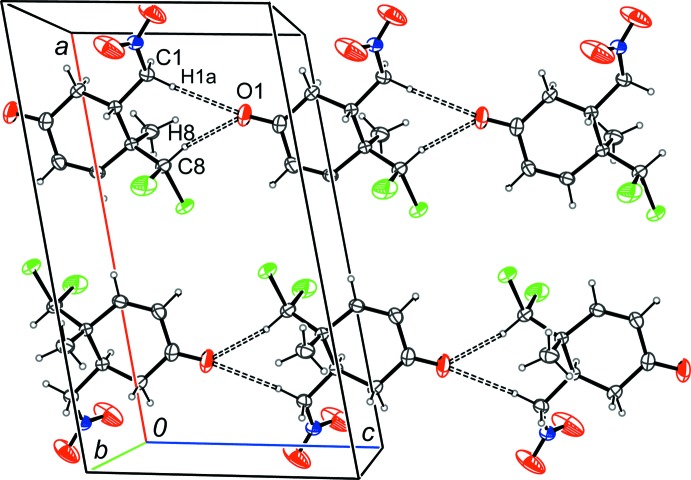
Segment of the crystal packing. A bifurcated C—H···O hydrogen bond connects the molecules into chains extended along c axis.

## supplementary crystallographic information

### 1. Comment

4-Dichloromethyl-4-methylcyclohexa-2,5-dienone, as a conjugated enone, readily undergo Michael reaction with carbanions, giving synthetically valuable polyfunctionalized products (Wenkert *et al.*, 1969). Utilizing this reaction, some natural products (Stefanović *et al.*, 1983), as well as some bioactive compounds (Solujić *et al.*, 1991; 1999) were successfully synthesized. We report now on synthesis of the title compound (I) by the same reaction using carbanion obtained from nitromethane.

The crystal structure of (I) is shown in Figure 1. None of the oxygen atoms of the nitro group is involved in hydrogen bonding. Similarly, two chlorine atoms also remain without the appropriate intermolecular donor, while there are two bent C—H···Cl intramolecular contacts shorter then the sum of van der Waals radii for H and Cl atoms [C6—H6a = 0.97, H6···Cl1 = 2.76 Å, C6—H6···Cl1 =106 °; C2—H2 = 0.98, H2···Cl2 = 2.66 Å, C2—H2···Cl2 = 112 °]. The most significant interaction in the crystal structure is a bifurcated C—H···O hydrogen bond [C8—H8 = 0.98; H8···O3^i^ = 2.24 Å; C8—H8···O3 = 164° and C1—H1a = 0.97; H1a···O3^i^ = 2.56 Å; C1—H1a···O3 = 164°] (symmetry code: i = *x*, *y*, *z* - 1)] which connects the molecules into chains extended along the *c* axis (Figure 2).

### 2. Experimental

Following the literature protocol (Wenkert *et al.*, 1969), to freshly prepared sodium methoxide in methanol a nitromethane solution of 4-(dichloromethyl)-4-methylcyclohex-2,5-dienone in dry methanol was added dropwise. After one hour stirring of the obtained solution, the solvent was evaporated and the rest quenched with diluted hydrochloric acid. The obtained mixture was extracted with toluene, the organic layer dried overnight (anh. sodium sulfate) and the solvent evaporated. The crude solid was recrystallized from hot toluene to give pure 4-(dichloromethyl)-4-methyl-5-(nitromethyl)cyclohex-2-enon.

### 3. Refinement

All H atoms were placed at geometrically calculated positions and included in the refinement in the riding model approximation, with C—H lengths of 0.93 (aromatic CH), 0.96 (CH_3_), 0.97 (CH_2_), and 0.98 Å (CH). *U*_iso_ of the H atoms were set at 1.5*U*_eq_ of the parent C for the methyl group and at 1.2*U*_eq_ otherwise.

### Crystal data

**Table:**

C_9_H_11_Cl_2_NO_3_	*F*(000) = 520
*M**_r_* = 252.09	*D*_x_ = 1.496 Mg m^−^^3^
Monoclinic, *P*2_1_/*c*	Cu *K*α radiation, λ = 1.54180 Å
Hall symbol: -P 2ybc	Cell parameters from 927 reflections
*a* = 13.8922 (7) Å	θ = 4.2–70.2°
*b* = 10.4531 (9) Å	µ = 5.14 mm^−^^1^
*c* = 7.8696 (5) Å	*T* = 293 K
β = 101.682 (6)°	Prismatic, colourless
*V* = 1119.12 (13) Å^3^	0.11 × 0.10 × 0.05 mm
*Z* = 4	

### Data collection

**Table:**

Agilent Gemini S diffractometer	2160 independent reflections
Radiation source: Enhance (Cu) X-ray Source	1674 reflections with *I* > 2σ(*I*)
Graphite monochromator	*R*_int_ = 0.016
Detector resolution: 16.3280 pixels mm^-1^	θ_max_ = 72.7°, θ_min_ = 5.3°
ω scans	*h* = −16→17
Absorption correction: multi-scan (*CrysAlis PRO*; Agilent, 2013)	*k* = −12→7
*T*_min_ = 0.288, *T*_max_ = 1.000	*l* = −9→9
4083 measured reflections	

### Refinement

**Table:**

Refinement on *F*^2^	Primary atom site location: structure-invariant direct methods
Least-squares matrix: full	Secondary atom site location: difference Fourier map
*R*[*F*^2^ > 2σ(*F*^2^)] = 0.073	Hydrogen site location: inferred from neighbouring sites
*wR*(*F*^2^) = 0.214	H-atom parameters constrained
*S* = 1.13	*w* = 1/[σ^2^(*F*_o_^2^) + (0.0912*P*)^2^ + 0.9148*P*] where *P* = (*F*_o_^2^ + 2*F*_c_^2^)/3
2160 reflections	(Δ/σ)_max_ < 0.001
137 parameters	Δρ_max_ = 0.44 e Å^−^^3^
0 restraints	Δρ_min_ = −0.46 e Å^−^^3^

### Special details

**Table:**

Experimental. Empirical absorption correction using spherical harmonics, implemented in SCALE3 ABSPACK scaling algorithm. 'CrysAlisPro (Agilent Technologies, 2013)'

### Fractional atomic coordinates and isotropic or equivalent isotropic displacement parameters (Å^2^)

**Table:**

	*x*	*y*	*z*	*U*_iso_*/*U*_eq_	
Cl1	0.42318 (8)	0.42826 (19)	0.16338 (17)	0.1132 (6)	
Cl2	0.37776 (11)	0.67949 (16)	0.2702 (2)	0.1229 (7)	
N1	0.0336 (2)	0.6681 (4)	0.1987 (4)	0.0681 (9)	
O1	0.0509 (4)	0.7541 (5)	0.2925 (9)	0.184 (3)	
O2	−0.0380 (4)	0.6677 (7)	0.1028 (8)	0.195 (3)	
C1	0.1041 (3)	0.5609 (4)	0.1953 (5)	0.0634 (9)	
H1A	0.1335	0.5699	0.0942	0.076*	
H1B	0.0686	0.4804	0.1849	0.076*	
C2	0.1853 (2)	0.5576 (3)	0.3579 (4)	0.0521 (8)	
H2	0.2108	0.6447	0.3804	0.063*	
C3	0.1430 (3)	0.5148 (5)	0.5143 (5)	0.0699 (11)	
H3A	0.0917	0.5739	0.5301	0.084*	
H3B	0.1135	0.4309	0.4909	0.084*	
C4	0.2196 (4)	0.5091 (6)	0.6783 (5)	0.0854 (13)	
C5	0.3186 (3)	0.4765 (5)	0.6616 (5)	0.0719 (11)	
H5	0.3671	0.4682	0.7614	0.086*	
C6	0.3420 (2)	0.4579 (4)	0.5085 (5)	0.0605 (9)	
H6	0.4067	0.4358	0.5073	0.073*	
C7	0.2720 (2)	0.4699 (3)	0.3368 (4)	0.0506 (8)	
C8	0.3265 (3)	0.5283 (5)	0.2041 (5)	0.0746 (12)	
H8	0.2791	0.5396	0.0946	0.090*	
C9	0.2362 (3)	0.3356 (4)	0.2733 (6)	0.0744 (11)	
H9A	0.2912	0.2782	0.2889	0.112*	
H9B	0.2057	0.3394	0.1525	0.112*	
H9C	0.1895	0.3056	0.3387	0.112*	
O3	0.1993 (4)	0.5259 (7)	0.8183 (4)	0.165 (3)	

### Atomic displacement parameters (Å^2^)

**Table:**

	*U*^11^	*U*^22^	*U*^33^	*U*^12^	*U*^13^	*U*^23^
Cl1	0.0604 (7)	0.1947 (17)	0.0927 (9)	0.0257 (8)	0.0351 (6)	−0.0027 (9)
Cl2	0.0976 (10)	0.1148 (12)	0.1631 (15)	−0.0342 (8)	0.0423 (9)	0.0388 (10)
N1	0.0521 (17)	0.086 (2)	0.0644 (18)	0.0131 (16)	0.0081 (14)	0.0057 (17)
O1	0.140 (4)	0.128 (4)	0.239 (6)	0.069 (3)	−0.066 (4)	−0.086 (4)
O2	0.124 (4)	0.229 (6)	0.187 (5)	0.107 (4)	−0.075 (4)	−0.105 (4)
C1	0.0504 (18)	0.080 (3)	0.058 (2)	0.0105 (17)	0.0056 (15)	−0.0045 (18)
C2	0.0448 (16)	0.063 (2)	0.0486 (16)	0.0008 (14)	0.0095 (13)	−0.0015 (14)
C3	0.0533 (19)	0.100 (3)	0.061 (2)	0.0049 (19)	0.0235 (16)	0.000 (2)
C4	0.083 (3)	0.125 (4)	0.051 (2)	0.006 (3)	0.0212 (19)	0.007 (2)
C5	0.066 (2)	0.097 (3)	0.0485 (19)	−0.004 (2)	0.0008 (16)	0.0104 (19)
C6	0.0432 (16)	0.076 (2)	0.060 (2)	−0.0024 (15)	0.0035 (14)	0.0088 (17)
C7	0.0409 (15)	0.063 (2)	0.0481 (16)	−0.0011 (13)	0.0105 (12)	−0.0021 (14)
C8	0.0508 (19)	0.115 (3)	0.060 (2)	0.004 (2)	0.0181 (16)	0.013 (2)
C9	0.060 (2)	0.069 (2)	0.092 (3)	0.0047 (18)	0.0090 (19)	−0.020 (2)
O3	0.131 (3)	0.317 (8)	0.0541 (19)	0.061 (4)	0.035 (2)	0.004 (3)

### Geometric parameters (Å, º)

**Table:**

Cl1—C8	1.782 (4)	C3—H3B	0.9700
Cl2—C8	1.768 (5)	C4—O3	1.204 (5)
N1—O2	1.119 (5)	C4—C5	1.448 (6)
N1—O1	1.157 (5)	C5—C6	1.324 (5)
N1—C1	1.493 (5)	C5—H5	0.9300
C1—C2	1.525 (5)	C6—C7	1.502 (5)
C1—H1A	0.9700	C6—H6	0.9300
C1—H1B	0.9700	C7—C8	1.536 (5)
C2—C3	1.534 (5)	C7—C9	1.538 (5)
C2—C7	1.549 (5)	C8—H8	0.9800
C2—H2	0.9800	C9—H9A	0.9600
C3—C4	1.498 (6)	C9—H9B	0.9600
C3—H3A	0.9700	C9—H9C	0.9600
			
O2—N1—O1	118.3 (4)	C6—C5—C4	122.0 (3)
O2—N1—C1	118.8 (4)	C6—C5—H5	119.0
O1—N1—C1	122.8 (4)	C4—C5—H5	119.0
N1—C1—C2	112.3 (3)	C5—C6—C7	124.9 (3)
N1—C1—H1A	109.2	C5—C6—H6	117.5
C2—C1—H1A	109.2	C7—C6—H6	117.5
N1—C1—H1B	109.2	C6—C7—C8	109.0 (3)
C2—C1—H1B	109.2	C6—C7—C9	108.9 (3)
H1A—C1—H1B	107.9	C8—C7—C9	108.2 (3)
C1—C2—C3	110.0 (3)	C6—C7—C2	109.2 (3)
C1—C2—C7	112.5 (3)	C8—C7—C2	109.8 (3)
C3—C2—C7	110.2 (3)	C9—C7—C2	111.6 (3)
C1—C2—H2	108.0	C7—C8—Cl2	112.3 (3)
C3—C2—H2	108.0	C7—C8—Cl1	112.5 (3)
C7—C2—H2	108.0	Cl2—C8—Cl1	107.7 (2)
C4—C3—C2	112.5 (3)	C7—C8—H8	108.0
C4—C3—H3A	109.1	Cl2—C8—H8	108.0
C2—C3—H3A	109.1	Cl1—C8—H8	108.0
C4—C3—H3B	109.1	C7—C9—H9A	109.5
C2—C3—H3B	109.1	C7—C9—H9B	109.5
H3A—C3—H3B	107.8	H9A—C9—H9B	109.5
O3—C4—C5	121.3 (4)	C7—C9—H9C	109.5
O3—C4—C3	121.7 (5)	H9A—C9—H9C	109.5
C5—C4—C3	116.9 (3)	H9B—C9—H9C	109.5

### Hydrogen-bond geometry (Å, º)

**Table:**

*D*—H···*A*	*D*—H	H···*A*	*D*···*A*	*D*—H···*A*
C8—H8···O3^i^	0.98	2.24	3.189 (5)	164
C1—H1a···O3^i^	0.97	2.56	3.503 (6)	164

Symmetry code: (i) *x*, *y*, *z*−1.
